# Retraction: Multi-modal deep learning methods for classification of chest diseases using different medical imaging and cough sounds

**DOI:** 10.1371/journal.pone.0351197

**Published:** 2026-06-23

**Authors:** The PLOS One Editors

The *PLOS One* Editors retract this article [1] due to concerns about compromised peer review.

In addition, in Fig 2 of this article [1], the top two rows in the columns labeled Covid-19, pneumonia, tuberculosis, lung cancer, edema, and consolidation lung appear similar to the COVID-19 Omicron and pneumonia rows in Fig 2 of [2, retracted in 3] and the Dataset 1 and Dataset 2 rows in Figure 1 of [4], despite representing different conditions.

TA did not agree with the retraction. HM either did not respond directly or could not be reached.
